# Emil Kraepelin: A pioneer of scientific understanding of psychiatry and psychopharmacology

**DOI:** 10.4103/0019-5545.64591

**Published:** 2010

**Authors:** Andreas Ebert, Karl-Jürgen Bär

**Affiliations:** 1 Department of Psychiatry, Psychotherapy and Psychosomatic Medicine, Ruhr-University, Bochum, Germany; 2 Department of Psychiatry, Psychotherapy and Philosophenweg: 3, University Hospital, Jena, Germany

Emil Kraepelin was an influential German psychiatrist who lived in the late 19^th^ and the early 20^th^ century. His work had a major impact on modern psychiatry and its understanding of mental illnesses based on natural scientific concepts.

Kraepelin was born in 1856 in the small town of Neustrelitz in Northern Germany. Early residencies of his life were Leipzig and Würzburg, where he studied medicine as well as Munich, where he began his career as a psychiatrist before moving on to Leipzig again. Wilhelm Wundt, a well-known psychologist, philosopher and physiologist who met Kraepelin there, became a particularly influential person in Kraepelin’s life. Kraepelin worked with enthusiasm in Wundt’s laboratory in Leipzig where his dedication to scientific research became obvious. As a result of spending most of his time in the laboratory, he lost his position as a physician because of neglect of clinical work. After this short stay in Leipzig and his qualification as a university teacher, Kraepelin worked as a visiting professor for five years in Dorpat (Estonia) and later in Heidelberg (now as a regular professor).[1] In 1903, he moved to Munich where he founded the Department of Psychiatry of the University. It was his laboratory in which Alois Alzheimer studied the underlying causes of Alzheimer dementia. Emil Kraepelin died in 1926 in Munich after having dedicated his last years to the work on his psychiatric textbook (*Lehrbuch der Psychiatrie*) and the development of the *Deutsche Forschungsanstalt für Psychiatrie* (German Research Institute for Psychiatry).

One of the most important achievements of Emil Kraepelin was the connection of pathogenesis and manifestation of psychiatric disorders.[2] In opposition to the leading theories of his time, Kraepelin did not believe that certain symptoms were characteristic for specific illnesses. Clinical observation led him to the hypothesis that specific combinations of symptoms in relation to the course of psychiatric illnesses allow one to identify a particular mental disorder. Today his concept of endogenous psychosis is regarded as Kraepelin’s main achievement. He differentiated between ‘dementia praecox’ and ‘manic depression’ as the two forms of psychosis. Kraepelin considered ‘dementia praecox’ (which is nowadays known as schizophrenia) as a biological illness caused by anatomical or toxic processes.[3] The reason for this denomination as dementia was that to Kraepelin; schizophrenia was a progressive neurodegenerative disease, which automatically resulted in irreversible loss of cognitive functions. In contrast, he described manic depression as an episodic disorder, which does not lead to permanently impaired brain function. In 1911, the Swiss psychiatrist Eugen Bleuler revised this idea, renaming ‘dementia praecox’ to schizophrenia. Nevertheless, the separation of affective disorders from schizophrenic psychosis as two distinct entities formed the basis for the understanding of psychiatric illnesses for more than a century. Over the last years both entities have been more and more regarded rather as a continuum than as two entirely distinct forms.[4] However, both the International Classification of Diseases (WHO) as well as APA’s DSM-Classification still rely on Kraepelin’s concept.

Although the dichotomic concept of psychosis is the best-known part of Kraepelin’s psychiatric work, there are a lot more achievements to his credit. He pioneered in the field of psychopharmacological research, which was uncommon in his days. For instance, he combined testing subjects with substances like alcohol, caffeine and chloroform with psychological tests.[5] One of the most problematical issues about Kraepelin is his generalization of psychiatric findings to social and political contexts. For example, socialists and opponents of World War I were judged to be mentally ill by him. He also theorized about frequent genetic predispositions for psychiatric disorders in Jews.[6] In addition to that, Kraepelin did not become known as an empathetic psychiatrist during his daily work with patients; in his opinion it was more important to observe than to listen to patients’ words.[7] This can also be seen as part of his struggle for objective observation and evidence. A matter of personal importance to him was his refusal not only of the abuse of alcohol, but also of alcohol in general. A possible explanation for this strong dislike might be that Kraepelin’s father suffered from alcoholism, which severely affected his relationship to him.[7] Concerning psychotherapy, Kraepelin was in favor of a supportive approach.[8] Although Kraepelin and Sigmund Freud shared the same year of birth, in professional matters they can be regarded as antipodes. This signifies that with regard to Freud’s psychoanalysis, Kraepelin always kept a highly critical point of view. Especially dream interpretation provoked his resistance; he judged psychoanalysis as not sufficiently based on scientific principles. Those different points of view still cause controversies nowadays when it comes to integrate natural scientific and psychodynamic understandings in psychiatry. In conclusion, he was a great thinker and started the scientific understanding of mental illness in a real sense.
